# Recombinant Baculovirus Associated with Bilosomes as an Oral Vaccine Candidate against HEV71 Infection in Mice

**DOI:** 10.1371/journal.pone.0055536

**Published:** 2013-02-04

**Authors:** Balraj Premanand, Mookkan Prabakaran, Tanja K. Kiener, Jimmy Kwang

**Affiliations:** 1 Animal Health Biotechnology, Temasek Life Sciences Laboratory, National University of Singapore, Singapore, Singapore; 2 Department of Microbiology, Faculty of Medicine, National University of Singapore, Singapore, Singapore; University of Utah School of Medicine, United States of America

## Abstract

**Background:**

Human enterovirus 71 (HEV71) is one of the major pathogen responsible for hand, foot and mouth disease (HFMD). Currently no effective vaccine or antiviral drugs are available. Like poliovirus, EV71 is transmitted mainly by the feco-oral route. To date the majority of the studied EV71 vaccine candidates are administered parenterally. Injectable vaccines induce good systemic immunity but mucosal responses are often unsatisfactory, whereas mucosal vaccines provide both systemic and mucosal immunity. Therefore, oral immunization appears to be an attractive alternative to parenteral immunization.

**Methodology/Principal Findings:**

In this report, we studied the efficacy of an orally administered vaccine candidate developed using recombinant baculovirus displaying VP1 (Bac-VP1) in a murine model. Gastrointestinal delivery of Bac-VP1 significantly induced VP1-specific humoral (IgG) and mucosal (IgA) immune responses. Further, we studied the efficacy of the Bac-VP1 associated with bilosomes and observed that the Bac-VP1 associated with bilosomes elicited significantly higher immune responses compared to bilosomes non-associated with Bac-VP1. However, mice immunized subcutaneously with live Bac-VP1 had significantly enhanced VP1 specific serum IgG levels and higher neutralizing antibody titers compared with mice orally immunized with live Bac-VP1 alone or associated with bilosomes.

**Conclusion:**

Bilosomes have been shown to possess inherent adjuvant properties when associated with antigen. Therefore Bac-VP1 with bilosomes could be a promising oral vaccine candidate against EV71 infections. Thus, Bac-VP1 loaded bilosomes may provide a needle free, painless approach for immunization against EV71, thereby increasing patient compliance and consequently increasing vaccination coverage.

## Introduction

Human enterovirus 71 (EV71) is a positive-stranded RNA virus belonging to the Enterovirus genus of the Picornaviridae family. EV71 has emerged as the most important neurotropic virus in young children after poliovirus [1]. Since 1997, EV71 infection has gained new significance with an increasing number of cases. Episodes caused by various strains of EV71 continue to reappear in countries such as Thailand, China and Vietnam [2]. The expanding geographic distribution of EV71 infections with recent outbreaks in Singapore indicates that more human populations are at risk [3]. Currently there are no effective vaccines or antivirals. Hence, developing vaccines is considered the best way to constrain the spread of EV71 infection. VP1 is thought to be mainly responsible for the attachment of virus to target cells [4] and hence harbours the main antigenic determinant for virus neutralization [5]–[6]. In our previous study, intramuscular (i.m.) or subcutaneous (s.c.) immunization of recombinant baculovirus surface displayed VP1 induced cross-neutralization activity against EV71 strains [7]–[8]. A passive protection study also showed that sera from the vaccinated mice protected six days old mice against EV71-B4 (5865/SIN/00009) infection [7]–[8]. Viral, bacterial or parasitic pathogens, including EV71, mostly initiate infection via the mucosal surfaces, and they spread via direct feco-oral route. However, the majority of the studied EV71 vaccine candidates are administered either subcutaneously or intramuscularly which stimulates only humoral immune responses [9]–[14]. Hence, oral vaccination should be considered as viable option to stimulate both systemic and mucosal immune response [15]. Previously Chiu et al. (2006) reported that oral vaccination of mice with *Salmonella*-based VP1 vaccines induced both humoral and cellular immune responses against EV71 but mucosal responses were not investigated [16].

There is a limitation for oral immunization in humans due to the lack of safe mucosal adjuvants. Most of the currently available adjuvants used in animal models are not acceptable for use in humans [17]–[20]. Safe adjuvants and optimum antigen delivery strategies are capable of inducing effective local protective immune responses against EV71. To date, a variety of viral vectors for vaccine delivery has been reported. One of the most promising vectors is *Autographa Californica* multiple nucleopolyhedrovirus (AcMNPV), an enveloped double-stranded DNA virus which can drive the expression of foreign genes in mammalian cells without causing cytotoxic effects [21]. Oral administration of AcNPV displaying antigens has been shown to enhance humoral and mucosal immune responses in mice [22]. However, there could be a loss of vaccine antigens due to protein denaturation caused by the harsh intrinsic environment of the gastrointestinal tract [23]–[24]. This can be overcome by utilizing appropriate vaccine carrier systems. It has been demonstrated that antigens entrapped in bilosomes are protected from bile damage [25] and could initiate antigen-specific mucosal and systemic immune responses in mice [26]. Bilosomes are lipid-based vesicles, closely related to non-ionic surfactant vesicles (niosomes) [27]–[28] that consist of non-ionic amphiphiles forming a closed bilayer structure and incorporating bile salts. This system is compatible with a range of antigens [27]–[29]. In the current study, we determined whether the orally administered Bac-VP1 stimulates both systemic and mucosal immune responses. Also, we evaluated whether the protective potential of Bac-VP1 could be enhanced when associated with bilosomes.

## Materials and Methods

### Ethics Statement

All animal experiments were carried out in accordance with the Guidelines for Animal Experiments of the National Institute of Infectious Diseases (NIID). Experimental protocols were reviewed and approved by the Institutional Animal Care and Use Committee of the Temasek Life Sciences Laboratory, National University of Singapore, Singapore (IACUC approval number TLL-11–033). Mice were housed in individually ventilated cages (Tecniplast Sealsafe) provisioned with water and standard food, and they were monitored daily for health and condition. Total limb paralysis or more than 20% body weight loss was used as criterion for early euthanasia. The animals were euthanized by CO_2_ inhalation for five minutes.

### Viruses and Cells

Baculovirus, *Autographa californica* nuclear polyhedrosis virus (AcNPV), was propagated in *Spodoptera frugiperda* Sf9 II and Sf9 III cell lines (ATCC) which were grown at 27°C in serum-free medium SF-900 II and SF-900 III (Invitrogen), respectively. EV71 strains were grown in rhabdomyosarcoma (RD) cells in Dulbecco’s modified Eagle medium (DMEM) (Gibco, USA) at 37°C with 5% CO_2_. The 50% tissue culture infective dose (TCID_50_) was determined in Vero cells using the Reed and Muench formula [30]. Inactivated recombinant baculovirus and EV71 vaccines were prepared by treating with binary ethylenimine (BEI) as described previously [31]–[32]. Briefly, the virus was inactivated by 3.2 mM of binary ethylenimine (BEI) at 37°C for 48 h and the residual BEI was hydrolyzed in samples by the addition of 1 mol/L sterile Na-thiosulfate (Merck) solution at 10% of the culture volume. The complete loss of infectivity of the inactivated baculovirus was determined by inoculation into Sf9 cell monolayers, whereas inactivation of EV71 was determined by inoculation into RD cells and observation of absence of cytopathic effects for at least 7 days.

### Construction of Recombinant Baculovirus

The full-length open reading frame (ORF) of the VP1 gene of EV71 virus (C4 strain) was amplified from genomic RNA of Fuyang EV71-C4 strain (Genbank accession #EU703813) by RT-PCR using primers VP1-F (5′-GCATTCTGCCTTTGCGGATCTGCAGGGAGATAGGGTGGCAGATG) and VP1-R (5′- CATTTGCGCGTTGCAGTGCTCAAGAGTGGTGATCGCTGTG). In total five primers were designed to construct the novel expression cassette. Primers A (5′- TTTGGCGGCGGCGGCGCATTCTGCCTTTGCGGGAGATAGGGTGGCAGATGTAATTG) and B (5′- CATGATATGGCAAAGGAGGTCCATAGGATCCAAAGAGTGGTGATCGCTGTGCGAC) were used to amplify the entire full length VP1 gene and to introduce additional overlapping regions of the gp64 signal sequence at the N-terminus and H3N2 transmembrane region at the C-terminus of VP1. The resulting amplicons were purified and subjected to a second PCR using primers A and C (TTGGCAGGCCCACATGATGAACCCCAACAAAGCAACACAAAGCAAAAAACATGATATGGCAAAGGAAATCCA) to add the entire sequence of the H3N2 transmembrane domain to the C-terminus of VP1. Primers D (5′-AATCGGTCCGATGGTAAGCGCTATTGTTTTATATGTGCTTTTGGCGGCGGCGGCGCATTCTG) and E (5′- AATAAGCTTTTAATATTGTCTATTACGGTTTTGGCAGGCCCACATGATGAACC) were then used to introduce the entire region of the gp64 signal sequence at the N-terminus and the gp64 CTD (cytoplasmic domain) region at the C-terminus of the above amplicons. Primer D and Primer E were designed to add restriction enzymes sites RsrII and Hind III at both terminuses, which allowed the insertion of the fusion genes into the RsrII/HindIII site of the multiple cloning site of pFASTBacHTA. Finally, the *polh* promoter was substituted with the *ie1* promoter, amplified from white-spot syndrome virus DNA, using AccI and Rsr II restriction sites and the Primers F (5′-CCTACGTATCAATTTTATGTGGCTAATGGAGA) and G(5′-CGCGTCGACCTTGAGTGGAGAGAGAGCTAGTTATAA). The final construct was designated as Bac-VP1.

For the generation of recombinant baculoviruses, the construct was integrated into the baculovirus genome within DH10Bac (Invitrogen) by site-specific transposition using the Bac-To-Bac system (Invitrogen) as described before [33]. The recombinant bacmids were then transfected into Sf9 cells, and the budded virus particles released into the medium were harvested at 4 days post transfection.

### Confocal Assay to Detect Expression of VP1 in Insect Cells

To detect the immunofluoresence signals, the Sf-9 II cells were cultured on sterile cover slips (placed in 6-well plates) and infected at an MOI of 0.1. Two days after infection, the cells were fixed in 4% PFA for 1 h at 4°C, rinsed with phosphate-buffered saline (PBS, pH 7.4), and blocked with 2% bovine serum albumin for 30 min at 37°C. The cells were then incubated with the polyclonal primary antibody raised against bacterially expressed VP1 in guinea pig (1∶300) for 1 h at 37°C, followed by three PBS washes. The cells were then incubated with the secondary FITC-conjugated rabbit anti-guinea pig mAb (1∶100 dilutions, Dako) for 1 h at 37°C, followed by three PBS washes. The negative control cells were treated the same way. Plasma membrane staining was done according to the manufacturer’s instructions (CellMask™ Orange plasma membrane stain, Invitrogen) Protein localization was visualized using a confocal microscope (LSM 510, Zeiss, USA).

### Characterization of Baculovirus Displaying VP1

The virus particles were purified by two rounds of sucrose gradient ultracentrifugation following standard protocols and the viral titers were determined by PFU [34]. The purified baculoviruses were resuspended in PBS, mixed with Laemmli sample buffer and resolved by 12% SDS-PAGE. Fractions containing purified baculovirus were then transferred onto a nitrocellulose membrane and blocked with 5% non-fat milk in 1X PBS and 0.1% Tween 20 for 1 h at room temperature. The membrane was incubated with guinea pig anti-VP1 polyclonal antibodies at a dilution of 1∶100 and subsequently incubated with horseradish peroxidase (HRP)-conjugated rabbit anti- guinea pig antibodies (1∶3000, DAKO). The protein bands were visualized by the Amersham ECL plus Western blotting detection reagents (GE Healthcare, USA).

### Preparation of Recombinant Baculovirus (Bac-VP1) Loaded Bilosomes

Bile salt stabilized vesicles (bilosomes) were prepared as previously described [35] with some modifications. Briefly, sorbitan tristearate, cholesterol and dicetyl phosphate in a molar ratio of 7∶3:1 were dissolved in 10 ml chloroform in a round-bottom flask. The chloroform was removed under reduced pressure using a rotary evaporator to produce a thin film on the side of flask. The film was subsequently hydrated with 4 ml of PBS buffer pH 7.4. The lipid mixture was homogenised at 8500 rpm for 5 min and 1 ml sodium deoxycholate (Sigma-Aldrich Ltd., UK) in PBS buffer (100 mg/ml) was added (preheated to 60°C). The mixture was homogenised for another 2 min and then incubated at 30°C in a water bath. Inactivated or live Bac-VP1 (2000 µg of total protein) were added to the homogenate and the total volume was made up to 5 ml with PBS buffer. Bac-VP1 association was achieved by subjecting the suspension to five freeze-thaw cycles in liquid nitrogen. The bilosome suspension was centrifuged for 2 h at 200,000×g using a Beckman XL-90 ultracentrifuge (Beckman RIIC, UK) to remove any non-associated antigen. The vesicles were then re-suspended in 5 ml PBS buffer (pH 7.4). The amount of antigen associated with the bilosomes was quantified using a modified ninhydrin assay as described previously [36]. The amount of VP1 present in recombinant baculovirus associated with bilosomes was calculated as described previously [37]. Briefly, Odyssey infrared imager (LI-COR, Biotechnology) was used to calculate the amount of VP1 present on recombinant baculovirus associated with bilosomes. The recombinant VP1 (rVP1) expressed in bacterial system and purified with His-tag fusion partner was used as a standard. An anti-guinea pig VP1 polyclonal antibody at adilution of 1∶500 and a dilution of 1∶10,000 of donkey anti-guinea pig IRdye800CW IgG (LI-COR, Biotechnology) were used as primary and secondary antibody respectively. The membrane was developed and band intensities were analysed by Odyssey Application software version 1.2. The amount of VP1 displayed on Bac-VP1 was calculated by comparing with the rVP1 as standard control. The same experimental conditions were used for standard and samples and a correlation coefficient of >0.99 was obtained.

### Negative Staining

Transmission electron microscopy with negative staining was used to visualize the associated antigens present within the bilosomes formulation. Formvar/carbon-coated 200 mesh copper grids were glow discharged and a droplet of vesicle suspension applied. 2% phosphotungstic acid (Sigma, St. Louis, MO, USA) negative stain was added and the grids were immediately dried. The grid was examined by transmission electron microscopy (H-7500, Hitachi, Tokyo, Japan).

### Oral Immunization

Specific-pathogen-free female BALB/c mice (6 weeks old) were obtained from the Laboratory Animals Centre, National University of Singapore, and maintained at the Animal Holding Unit of the Temasek Life Sciences Laboratory. Prior to oral immunization, all mice were starved for 2 h and fed with sodium bicarbonate buffer (pH 9.6) to protect the antigens from the gastric acid. Ten mice per experimental group (n = 10/group) were immunized intragastrically by oral gavage on days 0, 7, and 21 with 200 µl of live Bac-VP1 or inactive Bac-VP1 (10^7^ PFU) associated or non-associated with bilosomes. In addition, a group of mice (n = 10) was subcutaneously immunized with 200 µl of Bac-VP1 and a subsequent booster dose was given on day 21. As positive control, a group of mice (n = 10) was immunized s.c. with 200 µl of inactive EV71 (C4) virus (10^7^ TCID_50_) with complete Freund’s adjuvant and the subsequent booster dose was administered using incomplete Freund’s adjuvant on day 21. As negative controls, mice (n = 10) were immunized with baculovirus wild type (Bac-wt) virus or PBS. Serum and intestinal lavage fluids were collected as described previously [38]. Briefly, the small intestine from each mouse was cut into 4- to 5-cm pieces and transferred to a glass tube. After addition of 1.0 ml of PBS, the tubes were vortexed gently for 30 s and centrifuged at 5,000 rpm for 10 min. The final supernatant was collected and used for the assay.

### Measurement of Anti-EV71 VP1-specific Antibodies by Indirect ELISA

The VP1-specific serum IgG and mucosal IgA levels were tested separately against purified recombinant VP1 antigen by indirect enzyme-linked immunosorbent assay (ELISA). Briefly, U bottom microtiter plates were coated with purified EV71 capsid protein VP1 in carbonate coating buffer (15 mM Na_2_CO_3_, 35 mM NaHCO_3_, pH 9.6). The plates were incubated at 4°C overnight and then incubated with 1% BSA in PBS with 0.05% tween20 (PBS-T) for 1 h at room temperature to prevent non-specific binding, 200 times diluted sera of different time points were added to the plates in duplicates and incubated for 1.5 h at room temperature. After three washes with PBS-T, 1000 times diluted horseradish peroxidase (HRP) conjugated goat anti-mouse immunoglobulins (DAKO) were added into each well. The reaction was developed by 100 µl TMB substrate (3, 3′, 5, 5′-etramethylbenzidine) and then terminated by 50 µl of 2 M H_2_SO_4_. The optical densities at 450 nm were determined using a microwell plate absorbance reader (Tecan, Switzerland).

### Virus Neutralization Titer (NT) Assay

The microneutralization test was performed according to a previously described protocol [33]. Briefly, RD cells were seeded in 96-well culture plates and cultured at 37°C to form a monolayer. Serial 2-fold dilutions of heat-inactivated (56°C for 45 min) serum samples were mixed separately with 100×50% tissue culture infective doses (TCID_50_) of EV71 virus and incubated at 37°C for 2 h, and the mixtures were added to the monolayer of RD cells in triplicate wells. After incubation for 5 days at 37°C, the neutralizing antibody titers were read as the highest dilution of sera that completely inhibited virus growth.

### Passive Protection Study in Mice

Mouse-adapted EV71-B4 strain was produced as described previously [7]. Briefly 1-day-old Balb/c mice were intraperitoneally (i.p.) injected with 10^7^ TCID50 of parental EV71- B4 strain (Genbank accession #AF316321.2). Five days later, the mice were euthanized and their brain tissues were dissected. After homogenization, the tissue lysates were collected, sterile filtered and used for further inoculation (i.p.) in to 1-day-old Balb/c mice. Six mouse brain passages later, the adapted virus caused 100% mortality in 6 day old mice. The protective immunity of vaccinated sera against EV71 infection was assessed in a mouse model of infection. 1-week-old BALB/c mice (n = 6) were intraperitoneally (i.p.) administered with vaccinated sera on day one post challenge with 5 MLD_50_ (Mouse lethal dose) of mouse adapted EV71 B4 strain HFM 41 (5865/SIN/00009). Fifty percent mouse lethal dose (MLD50) of the virus required for the experiments was predetermined. Mice from the positive control or negative control group were given an equal amount of vaccinated sera obtained from mice immunized with inactivated EV71 vaccine or Bac-wt or PBS. Survival rates and clinical scores of the mice were monitored daily until 21 days post infection.

### Quantitative Reverse Transcription Polymerase Chain Reaction (qRT-PCR) Assay

Brain tissues from the passively immunized mice were homogenized for total RNA extraction by Trizol reagent (Invitrogen). Extracted RNA (1 µg) was used for qRT-PCR using Quantifast SYBR Green RT-PCR kit (Qiagen, Hilden, Germany) according to the manufacturer’s protocol. 5′ UTR EV71 specific primers, i.e UTR-F-5′-TCCTCCGGCCCCTGAATG-3′and UTR-R-5′-GGACACCCAAAGTAGTCGGTTC -3′ were used for amplification. The qRT-PCR thermal cycling conditions were an initial incubation at 50°C for 12 min (reverse transcription), 95°C for 6 min (initial PCR activation step), followed by 40 cycles each of 95°C for 12 s (denaturation), 60°C for 30 s (combined annealing and extension), and 77°C for 15s. Melting curve data were collected from 50–95°C at a ramping of 1°C/5 s, and a finally cooling at 40°C was performed. The reaction was carried out using a Rotor-Gene-Q real time PCR cycler (Qiagen). The relative expression values were normalized to the expression value of the β-tubulin housekeeping gene. Serial ten-fold dilutions of recombinant plasmid DNA containing full-length EV71 genome were included to generate a standard curve for quantitative analysis.

### Statistical Analysis

The data were expressed as arithmetic mean ± standard deviation (SD). The unpaired two-tailed Student’s t-test was performed to determine the level of significance in the difference between means of two groups. The level of significance was expressed as P<0.05.

## Results

### Confirmation of VP1 Expression in Insect Cells and its Anchoring on the Baculoviral Envelope

The recombinant baculovirus (Bac-VP1) was constructed to express the VP1 on the baculoviral envelope by incorporationg a signal peptide and transmembrane domain with a CTD (cytoplasmic domain). Ideally, the gp64 signal sequence directs the translocation of the fusion protein to the insect cell plasma membrane during virus replication and is cleaved after protein anchoring thus exposing the VP1-N-terminus to the outer surface. The H3N2-HA transmembrane domain enables the protein to anchor into the plasma membrane, while the gp64 CTD mediates (1) the recognition of budding baculovirus nucleocapsid with the membrane-bound protein and (2) the incorporation of the anchored protein into the viral envelope. H3N2-HA expression and the position of the gp64 CTD was illustrated in our previous study [7].

**Figure 1:**
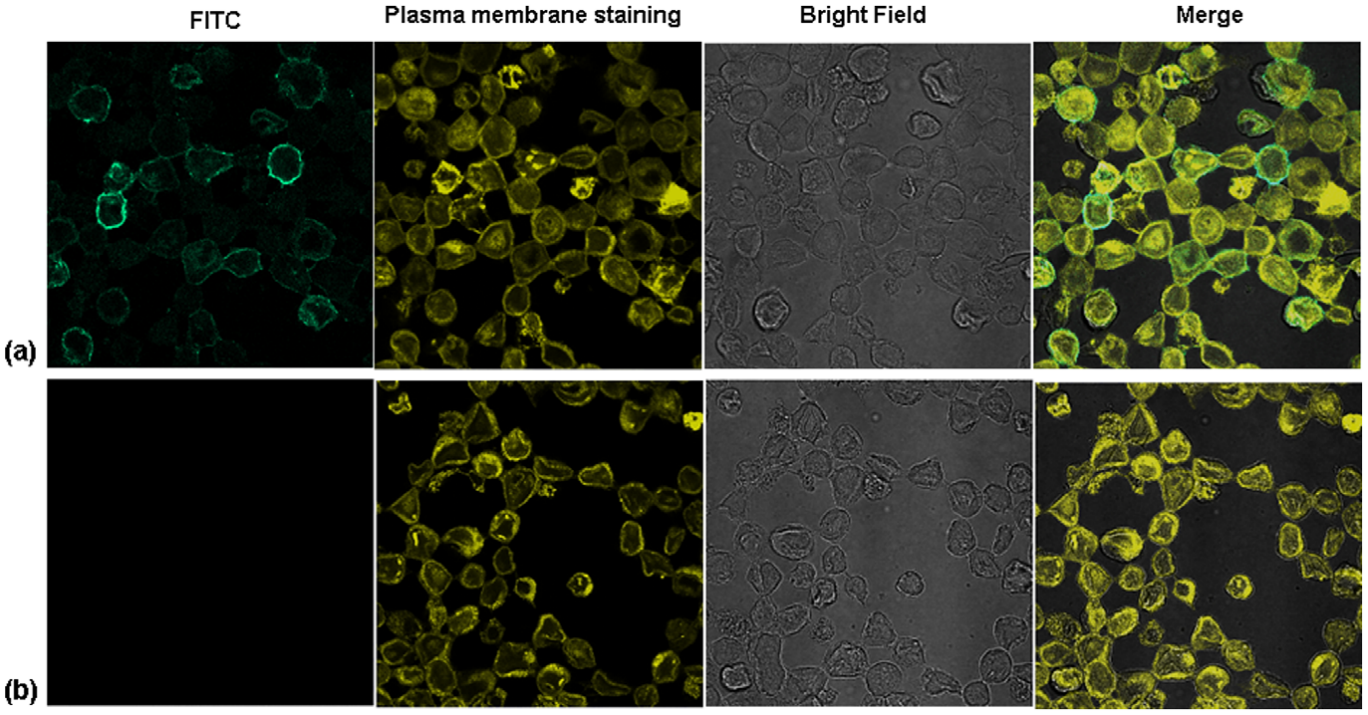
Anchoring of VP1 on the surface of infected Sf9 cells. (**a**) Confirmation of the anchoring of VP1 on the plasma membrane of Sf9-II cells infected with Bac-VP1. (b) No anchoring of VP1 was observed on the plasma membrane of Sf9- II cells infected with Bac-wt. The cells were cultured on sterile cover slips and infected at an MOI of 0.1. Cells were fixed with 4% PFA and blocked with 2% bovine serum albumin for 30 min at 37°C. VP1 was detected by a polyclonal primary antibody against bacterially expressed VP1 raised in guinea pig (1∶300 dilution; in house production) followed by a secondary FITC-conjugated rabbit anti-guinea pig mAb (1∶100 dilution; Dako). Plasma membrane staining was done using the CellMask™ Orange plasma membrane stain (Invitrogen).

**Figure 2:**
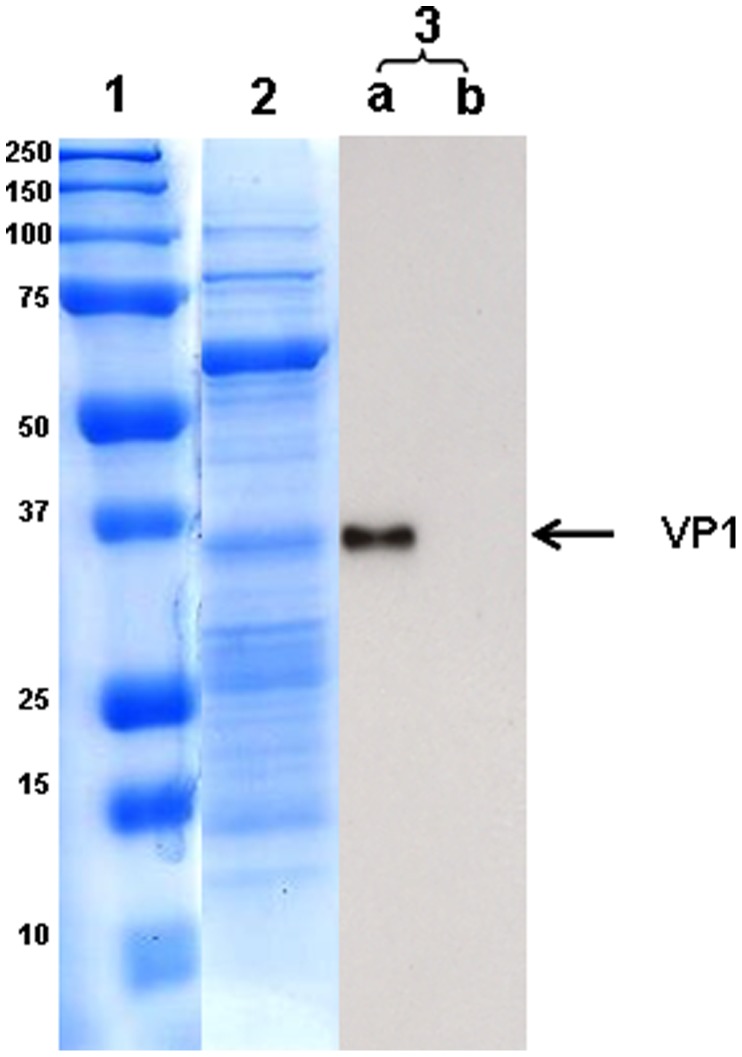
Confirmation of VP1 expression in purified baculoviruses by Coomassie staining and Western blotting. Sodium dodecyl sulfate-polyacrylamide gel electrophoresis (SDS-PAGE) of purified baculoviruses, showing the presence of VP1. Lane 1, prestained protein marker; lane 2, Coomassie staining; lane 3, Western blot using anti-VP1 guinea pig polyclonal antibody and respective secondary antibody (a) Bac-VP1, (b) Bac-wt.

To confirm the expression of VP1 in insect *Sf9-II* cells, the cells were infected separately with Bac-VP1 and as a control with wild type baculovirus. Confocal microscopic analysis using plasma membrane stain and VP1 polyclonal anti-guinea pig VP1 antibody (1∶500) followed by respective secondary FITC conjugated antibody revealed that VP1 expressed by the recombinant baculovirus was able to successfully translocate to the plasma membrane of infected insect cells. No signal was detected in Bac-wt infected cells (Figure 1). The merged photographs illustrate the co-localization of VP1 with the plasma membrane. Based on the co-localization of the VP1 with the plasma membrane, it was confirmed that VP1 was indeed translocated and anchored to the plasma membrane. To examine whether the anchored VP1 on the plasma membrane was successfully incorporated into the baculoviral envelope, purified baculoviruses were analyzed by Western blotting. VP1 protein was detected at a molecular weight of around 37 kDa (Figure 2) using anti-VP1 guinea pig polyclonal antibody followed by respective secondary antibody. No band was observed in wild type baculovirus preparations.

### Negative Staining of Bac-VP1 Associated with Bilosomes

**Figure 3:**
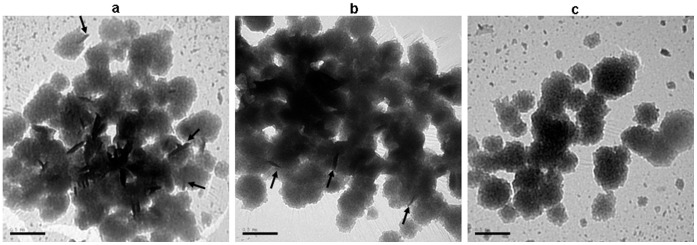
Negative staining of Bac-VP1 associated with bilosomes. (a) Bac-VP1 associated with bilosomes, (b) Inactive Bac-VP1 associated with bilosomes (c) Bilosomes only. Imaging was carried out using a transmission electron microscope JEM-1230 (Jeol). Arrows pointing to the successful association of Bac-VP1 with bilosomes.

Bilosomes were prepared from the mixture of sorbitan tristearate, cholesterol and dicetyl phosphate in 7∶3:1 molar ratio as described in materials and methods. Association of live or inactivated Bac-VP1 was achieved by addition the 2000 µg of total protein to the bilosomes, followed by five freeze-thaw cycles in liquid nitrogen. The amount of antigen associated with bilosomes was quantified using ninhydrin assay. The results showed that the amount of protein antigen associated with bilosomes was around 50% of the input. Bac-VP1 associated with bilosomes was visualized by negative staining using 2% phosphotungstic acid followed by transmission electron microscopy analysis. Based on the images of typical nucleocapsid structure of baculovirus associated with bilosomes, we confirmed that Bac-VP1 was successfully associated with bilosomes (Figure 3).

### Systemic Antibody Responses to Vaccine Candidates

**Figure 4:**
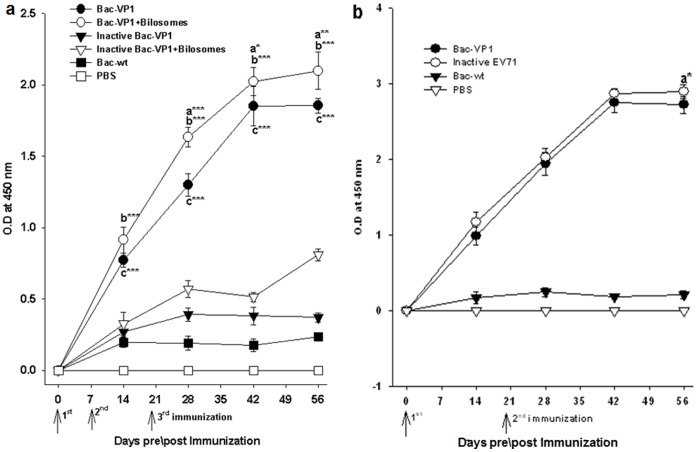
Measurement of VP1 specific IgG antibody response in the serum of immunized mice by indirect ELISA. (A) **Orally immunized mice:** Groups of mice (n = 10) were orally vaccinated three times on days 0, 7, and 21 with 200 µl containing live or inactivated recombinant baculovirus associated or non-associated with bilosomes. Bac-wt and PBS were given as negative controls. Each point represents the arithmetic mean value (n = 6) ±SD. *, P<0.05; **, P<0.01; ***, P<0.001 between live Bac-VP1 associated with bilosomes and live Bac-VP1 alone (a) or ***, P<0.001 between live Bac-VP1 associated with bilosomes and inactive Bac-VP1 associated with bilosomes (b) or ***, P<0.001 between live Bac-VP1 and inactive Bac-VP1 (c). (B) Subcutaneously immunized mice: Groups of mice (n = 10) were subcutaneously vaccinated two times on days 0 and 21 with 200 µl of live Bac-VP1 or inactivated EV71 or Bac-wt and PBS as negative controls. VP1-specific IgG antibody levels were determined by indirect ELISA. Each point represents the arithmetic mean value (n = 6) ± SD. *, P<0.05 between inactive EV71 and live Bac-VP1.

To explore whether the VP1 displayed on Bac-VP1 could serve as an immunogen, groups of BALB/c mice were immunized orally with live or inactive Bac-VP1 associated or non-associated with bilosomes and subcutaneously with live Bac-VP1. Immunizations with inactivated EV71, baculovirus wild type (Bac-wt) or PBS served as positive or negative controls respectively. At different time points (14, 28, 42 and 56 dpi) anti-sera were collected to determine the VP1-specific serum IgG levels. Mice orally immunized with live Bac-VP1 had significantly (P<0.001) enhanced VP1-specific IgG levels compared with inactive Bac-VP1 (Figure 4a). Also, the mice orally immunized with bilosomes associated live Bac-VP1 showed higher (P<0.001) VP1-specific IgG levels compared to inactive Bac-VP1 associated with bilosomes. Interestingly, sera of mice immunized with bilosomes associated Bac-VP1 showed significantly higher antibody levels (p<0.05) than mice immunized with Bac-VP1 alone (Figure 4a). Mice immunized subcutaneously with live Bac-VP1 induced VP1-specific IgG levels which were comparable to inactivated EV71 virus (Figure 4b). However, mice immunized subcutaneously with live Bac-VP1 or adjuvanted inactive EV71 showed significantly (P<0.001) higher VP1 specific IgG antibody response compared with mice orally immunized with live Bac-VP1 alone or associated with bilosomes (Figure 4a & 4b).

### Mucosal Immune Responses to Vaccine Candidates

**Figure 5:**
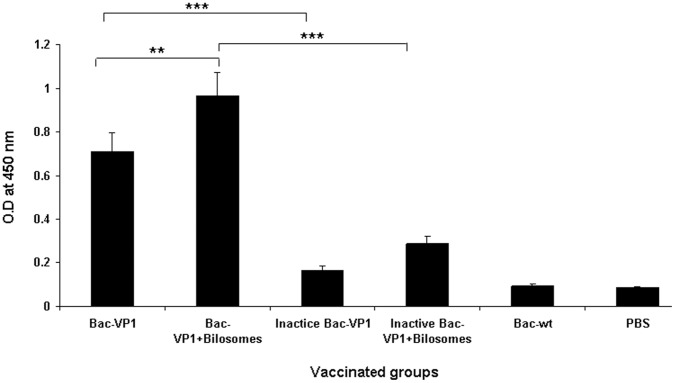
Measurement of VP1 specific mucosal IgA antibody response in orally immunized mice by indirect ELISA. Groups of mice (n = 10) were orally vaccinated three times on days 0, 7, and 21 with 200 µl containing live or inactivated recombinant baculovirus associated or non-associated with bilosomes or Bac-wt. Each point represents the arithmetic mean value (n = 6) ± SD. **, P<0.01 between live Bac-VP1 associated with bilosomes and live Bac-VP1 alone or ***, P<0.001 between live Bac-VP1 and inactive Bac-VP1 or ***, P<0.001 between bilosomes associated live Bac-VP1 and bilosomes associated inactive Bac-VP1.

To test whether Bac-VP1 can act as an immunogen and elicit a mucosal immune response, mice were immunized orally with live or inactive Bac-VP1 associated with bilosmes or non-associated with bilosmes. Immunizations with Bac-wt or PBS served as negative controls respectively. Mice were vaccinated orally on day 0 and boosted on day 7 and 21. Mucosal IgA antibody titers against VP1 were measured at day 56. The mice immunized with Bac-VP1 significantly (P<0.001) induced higher mucosal IgA levels than the mice immunized with inactive Bac-VP1. Again, the live Bac-VP1 associated with bilosomes showed higher (P<0.001) mucosal responses compared with inactive Bac-VP1 associated with bilosomes. The mucosal responses elicited by mice immunized with bilosomes associated Bac-VP1 were also significantly higher (P<0.01) than those of mice immunized with non-associated Bac-VP1(Figure 5).

### Serum Neutralization Titers of Vaccinated Mice

**Figure 6:**
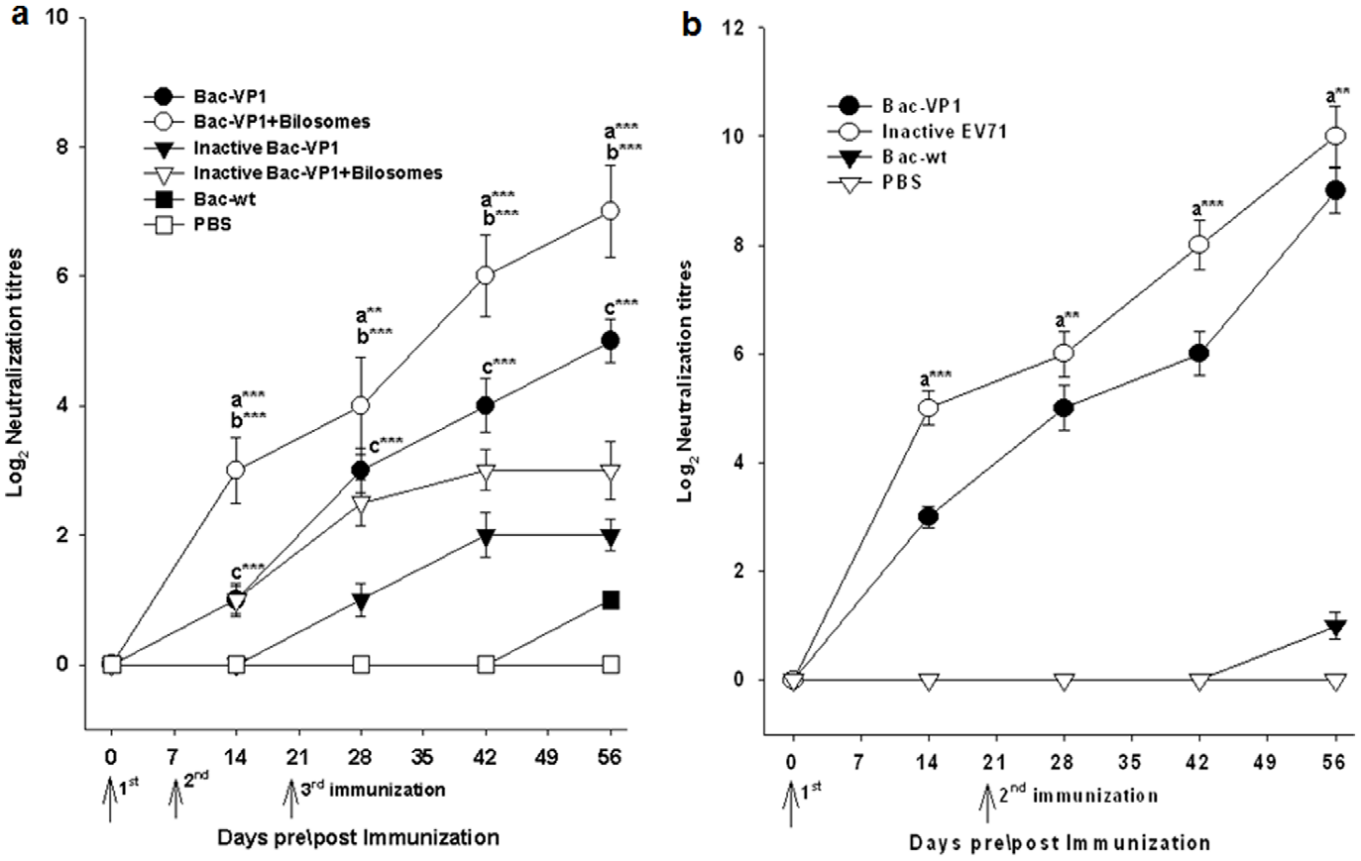
Neutralization antibody titers of vaccinated sera against homologous EV71 strain. Sera were collected from mice on days 0, 14, 28, 42, and 56. 25 µl of serial two-fold dilutions of sera were then mixed with 25 µl of 100 TCID50 of virus (Fuyang EV71, C4 virus) and incubated at 37°C for 2 h to neutralize infectious virus. The mixtures were then transferred to 96-well plates with more than 90% confluent monolayers of RD cells grown in DMEM containing 5% FBS. After incubation for 5 days at 37°C, the neutralizing antibody titers were read as the highest dilution of sera that completely inhibited virus growth. (A) Neutralization titers of orally vaccinated mice candidate’s sera. Each point represents the arithmetic mean value (n = 6) ±SD. **, P<0.01; ***, P<0.001 between live Bac-VP1 associated with bilosomes and live Bac-VP1 alone (a) or ***, P<0.001 between bilosomes associated live Bac-VP1 and bilosomes associated inactive Bac-VP1 (b) or ***, P<0.001 between live Bac-VP1 and inactive Bac-VP1 (c). (B) Neutralization titers of subcutaneously vaccinated mice candidate’s sera. Each point represents the arithmetic mean value (n = 6) ± SD. **, P<0.01; ***P<0.001 between inactive EV71 and live Bac-VP1.

**Figure 7:**
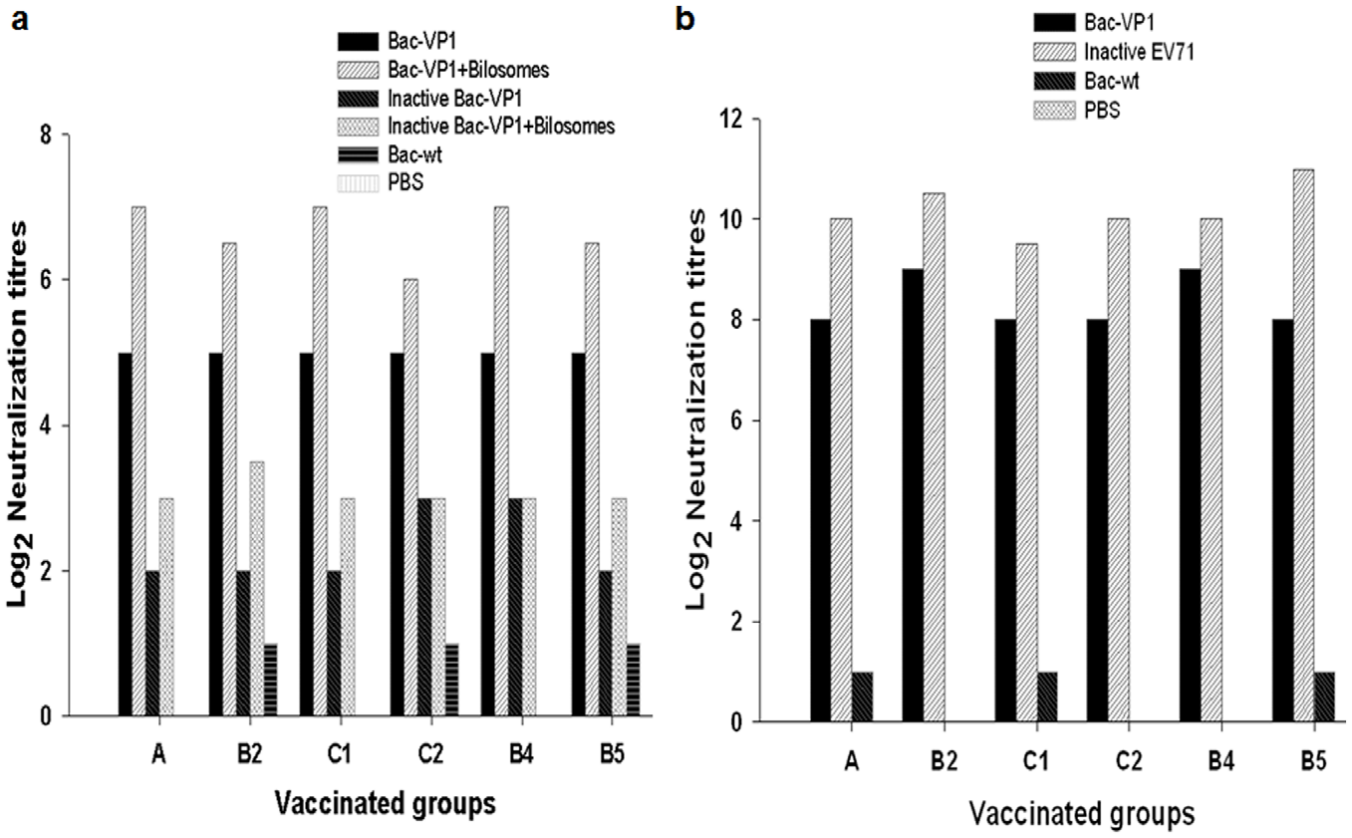
Neutralization antibody titers of vaccinated sera against heterologous EV71 strains. Sera were collected from mice on day 56. 25 µl of serial two-fold dilutions of sera were then mixed with 25 µl of 100 TCID50 of virus, and incubated at 37°C for 2 h to neutralize infectious virus. The mixtures were then transferred to 96-well plates with more than 90% confluent monolayers of RD cells grown in DMEM containing 5% FBS. After incubation for 5 days at 37°C, the neutralizing antibody titers were read as the highest dilution of sera that completely inhibited virus growth. (a) Neutralization titers of orally vaccinated mice candidate’s sera. (b) Neutralization titers of subcutaneously vaccinated mice candidate’s sera.

Next, the sera from vaccinated mice were analysed in an *in vitro* microneutralization assay to examine their ability to neutralize live EV71 strains in RD cells. Initially, neutralization assays were performed using the sera from different time points against homologous Fuyang strain (EV71-C4) to analyse their protective potential (Figure 6a, b). Sera from day 56 from both orally or subcutaneously immunized mice exhibited the maximum neutralizing activity against EV71 Fuyang strain. Later, we performed the neutralization assay using pooled sera from day 56 to verify whether these sera showed cross–protection against circulating heterologous EV71 strains (Table 1) (Fig. 7a, b). Results showed that sera from mice immunized orally with Bac-VP1 exhibited cross-neutralization activity at a maximum titer of 32 (2^5^). Interestingly the mice immunized orally with bilosomes associated Bac-VP1 exhibited a maximum neutralization activity of 128 (2^7^). However, the mice immunized orally with bilosome associated or non-associated inactive Bac-VP1 showed low neutralization activity of around 8 (Figure 7a). The sera from the group of mice immunized subcutaneously with Bac-VP1 exhibited the maximum neutralization activity of 512 (2^9^) compared to sera from the group of mice immunized orally with live Bac-VP1 alone or associated with bilosomes. The complete protection of RD cells using the sera of mice immunized with inactivated EV71 virus was observed to be a maximum of 2048 (2^11^) (Figure 7b). On the other hand, no neutralization activity was induced by Bac-wt or PBS antisera (<2^1^). This increase in neutralization efficiency of bilosomes associated Bac-VP1 compared to non-associated Bac-VP1 might be a direct result of the antigen protection by bilosomes in the gastrointestinal tract. Since neutralization assays were conducted against heterologous EV71 strains, these results also show that our construct elicits cross-protective antibodies.

**Table 1:** EV71 genotypes used in the *in vitro* microneutralization assay.

Isolate no.	Virus strain	Genotype	Origin	GenBank Ref.
1	BrCr	A	USA	U22521.1
2	7432/MS/87	B2	USA	U22522.1
3	Y90-3761	C1	Japan	AB433864
4	NUH0075/SIN/08	C2	Singapore	FJ172159.1
5	5865/SIN/00009	B4	Singapore	AF316321.2
6	NUH0083/SIN/08	B5	Singapore	FJ461781

### Passive Protection of Vaccinated Sera against Lethal EV71 Infection

**Figure 8:**
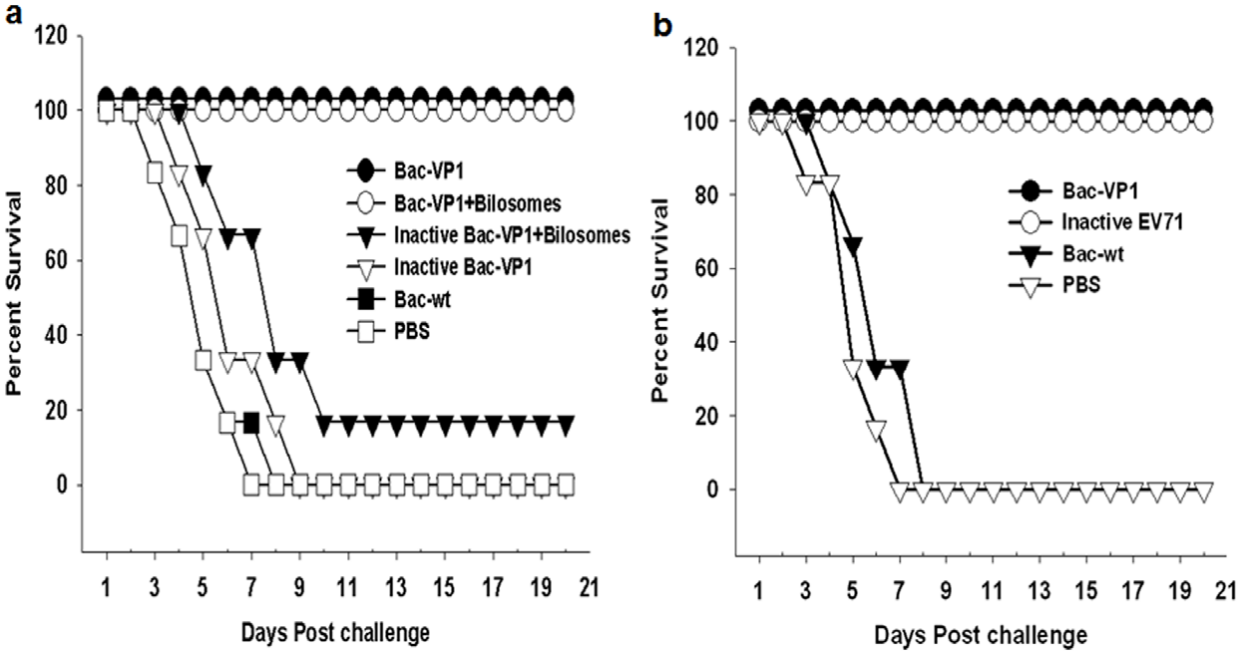
Passive protection of neonatal mice by vaccinated mice sera against mouse adapted lethal HEV71 infection. Six day old BALB/c mice were inoculated i.p. with mouse adapted EV71-B4 strain at a dose of 5 MLD50 per mouse. 24 h later, each mouse was passively immunized with sera obtained from the vaccinated adult mice. The control groups received sera from Bac-wt and PBS immunized mice. Mortality was monitored until 21 days post infection. (a) Passive protection study using sera from orally vaccinated mice (b) Passive protection study using sera from subcutaneously vaccinated mice.

**Figure 9:**
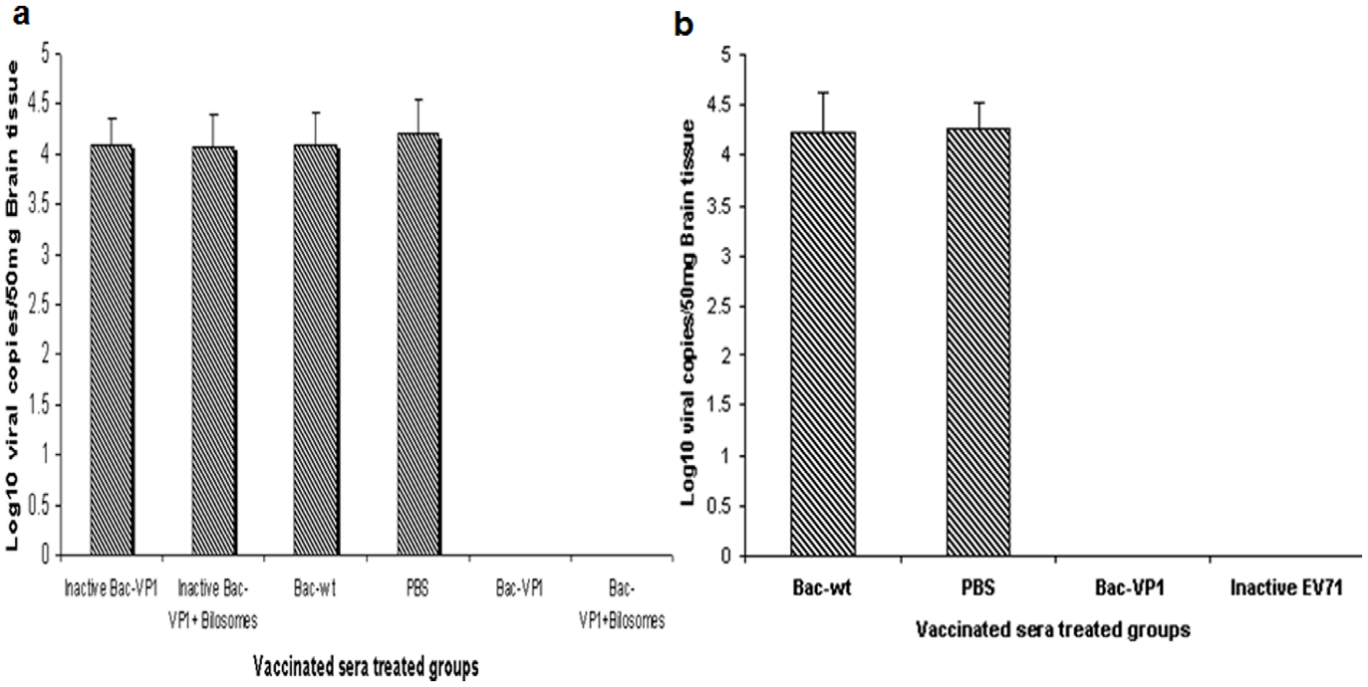
Measurement of viral copies in brain by real-time PCR. Mice were treated with orally or subcutaneously vaccinated sera on day 1 post challenge with 5 MLD_50_ (mouse lethal dose) of mouse adapted EV71 B4 strain HFM 41 virus. The viral loads were measured in the brain of dead and survived animals. (a) Mice treated with orally vaccinated mice sera (b) Mice treated with subcutaneously vaccinated mice sera. Each column represents the mean of triplicate assays with standard deviation.

The level of protective immunity induced by our vaccine constructs was determined in a suckling mouse model. Passive protective efficacy of the sera (day 56) from vaccinated mice were tested against 5 MLD_50_ of mouse-adapted EV71-B4 strain in 6 day old mice. Pups treated with sera from mice orally vaccinated with bilosome associated live Bac-VP1 had 100% protection against EV71 infection. However, pups treated with sera from mice orally vaccinated with bilosomes associated inactive Bac-VP1 had only 16.6% protection. Sera from mice orally vaccinated with live Bac-VP1 alone also conveyed 100% protection while mice that received sera from inactive Bac-VP1 vaccinated mice, all succumbed to the challenge (Figure 8a). Similarly, mice treated with sera from animals subcutaneously vaccinated with live Bac-VP1 or inactivated EV71 virus showed complete protection (Figure 8b). There was no protection conferred by the anti-Bac-wt or naïve sera. Further, real-time PCR was used to evaluate the viral RNA copies in the brain tissues of survivors and casualties. qRT-PCR was performed using 5′UTR EV71 specific primers. The viral titers were expressed as a measure of the number of copies of viral RNA. Pups treated with sera from live Bac-VP1 or bilosomes associated Bac-VP1 had no viral copies whereas pups treated with sera from inactive Bac-VP1 (associated or non-associated), Bac-wt or PBS immunized mice had copy numbers of ≥1×10^4^ copies/ml (Figure 9a, b).

## Discussion

Most human pathogens including EV71 initiate infection via the mucosal surfaces and therefore mucosal immunity is often required to achieve protection against these pathogens. Oral or intranasal administrations are the two main options for mucosal vaccination. Intranasal vaccines can have a detrimental effect on persons with asthma and other chronic pulmonary or cardiovascular disorders [39]. Therefore oral vaccines seem to be the safest alternative. Numerous vaccine delivery systems were identified for oral immunization [40] and reports have demonstrated that recombinant baculoviruses were also able to deliver genes of interest *in vivo* in animal models [41]–[43]. Previously, we have demonstrated that recombinant baculovirus was able to transduce a reporter gene into intestinal epithelial cells of orally immunized mice [22]. However, one of the major obstacles associated with the oral immunization approach is that the antigen in the vaccine formulation can be substantially degraded by gastric hydrochloric acid and proteolytic enzymes present in the gut [23]. Therefore more frequent doses of antigen might be needed to achieve a high level of immune responses. Protecting antigens from degradation by low pH and proteolysis can be achieved by packaging the antigen within particulate structures e.g. poly (D,L-lactide-co-glycolide) microspheres (PLG/PLGA) [44]–[46], immune stimulating complexes (ISCOMs), [47]–[48] or bilosome vesicles [49]. In the present study, we used bilosomes which are bile salt stabilized vesicles that are promising carriers for oral immunization of many antigens. Earlier reports have shown that oral administration of bilosomes incorporating an influenza subunit vaccine resulted in the generation of high titers of influenza virus-specific IgGs which were significantly greater than those achieved with the subunit vaccine alone [35]. Furthermore, oral delivery of influenza subunit antigens entrapped in bilosomes resulted in immune responses comparable with those achieved by conventional s.c. injection [35]. However, in our study the mice immunized subcutaneously with live Bac-VP1 significantly induced higher VP1 specific antibody response compared with mice orally immunized with either live Bac-VP1 alone or associated with bilosomes. Nevertheless, mice orally vaccinated with Bac-VP1 associated with bilosomes were able to induce stronger immune responses compared to live Bac-VP1 or inactive Bac-VP1associated with bilosomes as indicated by the high levels of VP1-specific IgG and mucosal IgA antibodies in ELISA. A microneutralization assay revealed that Bac-VP1 associated with bilosomes induced higher neutralizing antibodies against homologous and heterologous EV71 strains compared to non-associated Bac-VP1. The differences between the immune responses of the mice orally immunized with live Bac-VP1 and inactivated Bac-VP1 could be mainly due to two factors. First, inactivation might have altered the antigenicity of the displayed VP1 protein. Secondly, live Bac-VP1 or live Bac-VP1 associated with bilosomes could have been able to transduce cells efficiently and express additional VP1 antigen inside the cells as we have described in our previous article [7], whereas inactive Bac-VP1 was unable to transduce cells, due to the degradation of the genome caused by inactivation with BEI.

Interestingly, mice vaccinated parenterally with inactivated EV71 virus induced neutralizing antibodies at a titer of 2048 (2^11^) which was significantly higher than the neutralization titers of mice vaccinated with oral vaccine candidates. However, mice orally vaccinated with live Bac-VP1 alone or associated with bilosomes additionally induced VP1 specific mucosal IgA response compared to parenteral immunization that induces mainly humoral immune response. Protection against viral infectivity in an animal model has long been considered the best tool to test the efficacy of a vaccine candidate. One limitation of the vaccine efficacy study is the lack of a suitable mouse model of EV71 disease. Due to this, we have performed the passive protection assay in a suckling mouse model of EV71 infection. Sera from mice vaccinated orally with live Bac-VP1 alone or associated with bilosomes could completely protect pups against EV71 infection whereas sera from mice vaccinated orally with inactive Bac-VP1 associated or non-associated with bilosomes did not alleviate clinical symptoms of EV71 infected pups. Therefore, this study revealed that gastrointestinal delivery of VP1 by recombinant baculovirus elicited protective immunity against EV71 in mice. Moreover, the bilosomes associated Bac-VP1 efficiently induced humoral and mucosal immune responses against EV71. Besides its broad range of target cells coupled with its non-replicative nature [50], baculovirus possesses the following extra advantages: (i) recombinant baculoviruses are easy to construct and produce high viral titers by infecting insect cells in biosafety level 1 facilities; (ii) no pre-existing immunity against baculovirus has been reported in humans [51]; (iii) purification of baculovirus can be readily done by ultracentrifugation [34] or alternatively, by affinity chromatography columns [52].

In conclusion, oral vaccination is still considered the easiest way to deliver immunogens and also it is more acceptable to patients and reduces the need for highly trained personnel during mass immunization. Since bilosomes are produced from naturally occurring lipids and possess inherent adjuvant properties when associated with antigens, Bac-VP1 associated with bilosomes could be a viable option for an oral vaccine candidate against EV71 infection. Its advantages, together with its increased immunogenicity and protective efficacy in mouse models, make this recombinant baculovirus vaccine one of the safest vaccines; however it is necessary to investigate further the possible toxicity in animals before its use in clinical trials.
